# Langerhans Cell Histiocytosis Presenting in an Adult Patient With a Pleural Effusion

**DOI:** 10.7759/cureus.77541

**Published:** 2025-01-16

**Authors:** Jaime Loeza-Suárez, Paola Xanat Huertas-Castañeda, José María Toledo-Carrasquedo, Angel Trejo-Tejas, Alberto Monroy-Chargoy

**Affiliations:** 1 Internal Medicine, Hospital Juárez de México, Mexico City, MEX

**Keywords:** braf v600e mutation, case report, clinical pathology, different branches of internal medicine, hystiocitosis, rare cause of pleural effusion

## Abstract

Langerhans cell histiocytosis is a rare condition characterized by aberrant function and proliferation of the mononuclear phagocyte system. It can occur across all age ranges. This case report involves an adult female presenting with multisystem involvement and pleural effusion. A hypothesis suggests that chronic inflammatory states, resulting from cytokine production and the release of proinflammatory factors, alter vascular permeability, potentially leading to clinical manifestations such as fluid distribution abnormalities, including pleural effusion. Symptomatic multisystemic disease requires chemotherapy initiation. In this case, treatment could not be started due to unresolved empyema despite surgical intervention.

## Introduction

Langerhans cell histiocytosis (LCH) is a rare condition characterized by aberrant function and proliferation of the mononuclear phagocyte system [1]. It can occur at any age; however, it is most prevalent in individuals under 15 years of age. Its incidence in adults is approximately one to two cases per million per year [2].

LCH presents features of both reactive and neoplastic processes, with prognosis depending on presentation and organ involvement [3]. It can manifest as a single-organ disease with a tendency for self-resolution or as an aggressive, multisystemic condition with high mortality [3].

The most affected organs are the skeletal system and skin, although any organ can be involved. In adults, common clinical manifestations include diabetes insipidus and pulmonary nodules, particularly in smokers [4]. 

We present a case of an adult woman who presented with pleural effusion and fever.

## Case presentation

A 50-year-old woman presented with fever, diaphoresis, asthenia, adynamia, and myalgia in the lower limbs. One month later, she developed right and left cervical and left inguinal adenopathy, in conjunction with an unintentional weight loss of 15 kg. External evaluations revealed left pleural effusion and grade II microcytic hypochromic anemia, prompting referral to our hospital.

**Figure 1:**
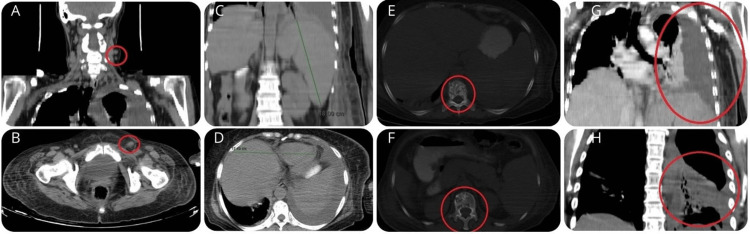
Clinical features A and B: Cervical and inguinal lymphadenopathy, with emphasis on the growth of the left node that was used for histological study. C: Splenomegaly, with a transverse measurement of 18 cm. D: Hepatomegaly measuring 17.48 cm. E and F: Lytic lesions in the thoracic vertebrae. G: Pleural effusion at hospital admission. H: Pleural effusion after drainage and decortication.

Physical examination identified cervical and left inguinal adenopathies, firm with irregular borders, non-tender, and measuring over 2 cm in diameter, along with pleural effusion and hepatosplenomegaly. Pleural fluid analysis revealed an exudate with infection by *Acinetobacter lwoffii*/*haemolyticus* (pansensitive) and *Staphylococcus haemolyticus *in two cultures. A computed tomography (CT) scan showed cervical and abdominopelvic adenopathies, osteolytic lesions, bilateral pleural effusion (predominantly left-sided), hepatosplenomegaly, and a hypodense splenic lesion (Figure 1).

**Figure 2:**
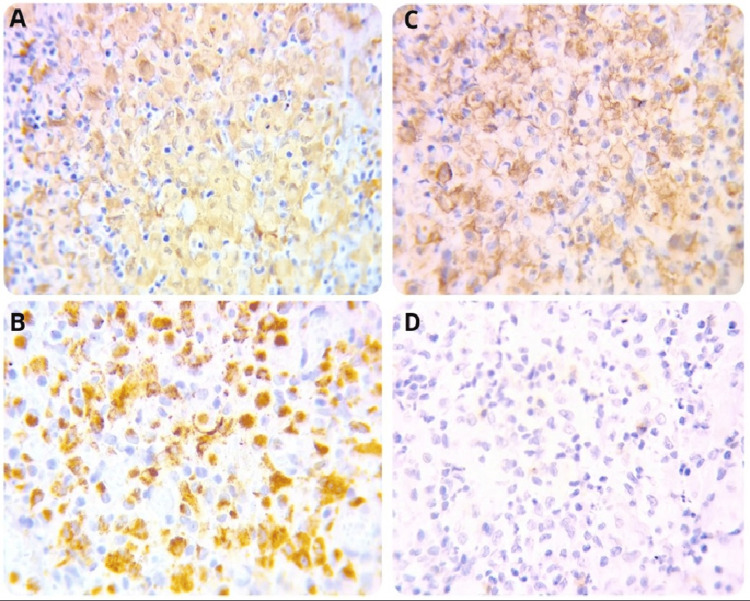
Immunohistochemistry Figure 2: A: S100 (400x). B: CD68 (400x). C: CD1A (400x). D: CD30 (400x). S100, CD68 and CD68 positive markers, CD30 negative. Interstitial Langerhans cells exhibit an immunohistochemical profile of CD1a+, S100+ according to the Histiocyte Society as strong evidence of diagnosis in almost 100% of cases. There is also the presence of some histiocytic markers such as CD68 with positivity in approximately 98% of cases, isolated expression of CD35 can occur. According to the literature, CD30 is only found in rare cases of true histiocytic tumors.

In the blood chemistry study, no abnormalities were observed (Table 1), blood count analysis showed leukocytosis with neutrophilia and regenerative anemia (Table 1). Inguinal lymph node biopsy with immunohistochemical analysis confirmed LCH, with positive S100, CD1a, and CD68 markers and negative CD30 (Figure 2).

**Table 1:** Laboratory findings Laboratory findings: No alterations in liver function tests or rest of blood chemistry. Regenerative anemia stands out without evidence of hemolysis.

	Value	Reference range
Lactic dehydrogenase	158 U/L	105-330 U/L
Direct bilirubin	0.33 mg/dl	0.01-0.30 mg/dl
Total bilirubin	0.73 mg/dl	0.01-1.0 mg/dl
Aspartate aminotransferase	27 U/L	10-39 U/L
Alanine aminotransferase	23 U/L	10-49 U/L
Leukocytes	24,100 U/L	5,200-12,400 U/L
Hemoglobin	9.0 gr/dl	12-18 g/dl
Corpuscular volume mean	79.9 fl	80-99 fl
Platelet count	412,000 U/l	130,000-400,000 U/L
Neutrophils	20,790 U/L	1,900-8,000 U/L
Lymphocytes	2,700 U/L	900-5,200 U/L
Reticulocytes	7.24%	0.5-1.50 %

Prior to the histopathological result, the diagnoses of lymphoma and mesothelioma were considered, although in the first case, there were no alterations in the blood count except for anemia. In the case of malignant mesothelioma, the patient had no history of exposure, and such a possibility would not have justified the osteolytic lesions or hepatosplenomegaly.

During hospitalization, antibiotic therapy with ciprofloxacin and ertapenem and drainage of empyema was started. However, the exudate continued to form, again isolating *Acinetobacter* in pleural fluid cultures, so surgical drainage and decortication of the left pleura were performed. Despite surgical treatment, the pleural effusion persisted, so it was not possible to start chemotherapy treatment. The patient was discharged with the best supportive care. The samples of pleura and pleural fluid were processed for pathological study, where no malignant cells were found.

## Discussion

Histiocytosis is a heterogeneous group of disorders characterized by the proliferation of macrophages and dendritic cells in various tissues [5]. Currently, they are classified into five groups according to the Histiocyte Society (2016): Langerhans cell (LC)-related, cutaneous and mucocutaneous, malignant, sporadic Rosai-Dorfman, and hemophagocytic lymphohistiocytosis [5]. Among these, LCH is the most common [6].

LCs are specialized antigen-presenting cells located in the epidermis, responsible for recognizing pathogens through the C-type lectin receptor langerin. Once internalized, these pathogens are sequestered in a specialized organelle known as the Birbeck granule [7]. LCH is defined by clonal expansion of myeloid precursors that differentiate into cells with high CD1a and langerin (CD207) expression [2,8].

Epidemiological data is scarce, especially for adults [9]. Although LCH can occur at any age, it primarily affects individuals under 15 years old, with a median age at diagnosis between 0 and 3 years and an incidence of 4-5 cases per million [10]. The male-to-female ratio is approximately 2:1 [11]. In adults, the incidence drops to 0.07 cases per million and is more common in young adults [11]. The disease appears sporadically, but familial cases have been reported in monozygotic or dizygotic twins [9].

Tissue macrophages (histiocytes) originate from hematopoietic myeloid progenitors that differentiate into monocytes, macrophages, and dendritic cells. In contrast, pathological LCs derive from immature myeloid precursors [6]. The etiology remains unclear, but most researchers agree that it results from immune dysfunction due to elevated cytokine levels in lesions compared to surrounding tissue [6]. Approximately 60% of cases exhibit the BRAF V600E mutation [12].

The BRAF-V600E (B-rapidly accelerated fibrosarcoma) mutation is an activating mutation leading to the constitutive activation of the RAS-RAF-MEK-ERK-MAPK pathway [12]. Under normal conditions, this pathway is activated by ligand binding to a receptor tyrosine kinase, leading to receptor phosphorylation, RAS activation, and downstream signaling through RAF, PI3K, and subsequent ERK activation, ultimately regulating key cellular survival processes. In LCH and other neoplasms harboring the BRAF-V600E mutation, ERK activation occurs independently of RAS or dimerization, promoting increased cellular survival, proliferation, and differentiation [13].

BRAF mutations have been implicated in prolonged inflammatory responses following infection, Bacillus Calmette-Guérin (BCG) vaccination, or tobacco exposure. Antigen stimulation induces interleukin-1 secretion by LCs, amplifying MAPK pathway activity. Physiological MAPK pathway activity is exacerbated by BRAF mutations [6].

LCH is considered a reactive disorder with a potential underlying neoplastic component [14], resulting from chronic inflammatory processes exacerbated by the BRAF mutation [14]. In cases not associated with the BRAF-V600E mutation, other mutations, such as ARAF, MAP2K1, and MEK1, may be implicated [14].

Reactive LCH can affect any organ [15]. It is classified based on the infiltrated tissue and the number of lesions into four subtypes: unifocal (single organ involvement), pulmonary unifocal (common in smokers, limited to the lungs), multifocal single-system (multiple lesions in one organ), and multisystemic (involvement of more than two organs) [3]. Localized disease generally follows a benign course with spontaneous remission, while multisystemic involvement often leads to aggressive disease despite various treatment modalities [16].

Clinical presentation depends on the affected organ. In bones, lytic lesions are observed, potentially involving the teeth, skull, pelvis, vertebrae, ribs, and extremities. CT imaging reveals lytic lesions affecting the cortex with a "punched-out" appearance [3]. Cutaneous manifestations include erythematous papular dermatosis with scaling and crusts on the thorax, back, abdomen, shoulders, scalp, and groin; oral mucosal, genital, and perianal lesions are less common [3]. Endocrine involvement may lead to pituitary dysfunction, often associated with diabetes insipidus, and can involve tumors in the pituitary, hypothalamus, and pineal gland [3]. Pulmonary involvement occurs in 40-50% of cases as a unique manifestation, with nodular or cystic lesions visible on HRCT. Liver and spleen involvement usually indicates multisystemic disease; early stages may present with hepatomegaly, nodules, or cholestasis, while advanced stages show severe cholestasis resembling rapidly progressing sclerosing cholangitis leading to hepatic failure [3].

This case is notable for involving an adult without tobacco exposure as a predisposing factor, presenting as a multisystemic disease (lymph nodes, bones, liver, and spleen). The disease onset was marked by respiratory symptoms and pleural effusion, without pulmonary involvement.

The patient presented with a complicated pleural effusion (exudative) diagnosed as empyema due to *Acinetobacter lwoffii*, unresponsive to antibiotics, and requiring surgical drainage and decortication. The effusion persisted despite surgery, and pleural pathology samples showed no malignancy.

In a study of 150 thoracoscopies with biopsies for exudative pleural effusions without a conventional diagnosis, malignancy was the most common etiology (56%, n = 73). Among benign causes, tuberculosis was predominant (83.7%, n = 37). The study also reported one case of exudative pleural effusion associated with LCH [17].

The chronic pro-inflammatory state, characterized by cytokine production and pro-inflammatory factors, is hypothesized to alter vascular permeability, clinically manifesting as fluid redistribution, including pleural effusion [16]. In other histiocytosis cases with pleural effusion, associations with thrombocytopenia, anemia, hypoalbuminemia, and elevated CD25 levels have been reported as risk factors [16]. The patient in this case exhibited anemia and hypoalbuminemia.

While the pathogenesis of pleural effusion in LCH is not fully understood, it is reasonable to attribute it to a pro-inflammatory state, as no evidence of pleural infiltration has been identified [16].

To diagnose LCH, a biopsy of the affected organ is required, followed by a panel of immunohistochemistry with CD207 (Langerin) and S100 expression. The cells are typically large, 15 to 25 micrometers, oval in shape, without the characteristic ramifications of dendritic cells, and the nucleus has a contour with a coffee bean-shaped groove [18]. Proliferations of LCs have generally been recognized as distinctive, but there has been debate about their neoplastic nature and their classification [19]. The expression of both CD1a and S100 protein is strong evidence for the diagnosis, as is the expression of some histiocytic markers such as CD68 and some focal and weak expression of lysozyme [19]. The immunophenotypic profile allows for easy distinction of LC neoplasms from interdigitating dendritic cell sarcoma [19]. The morphology of the typical form is easy to recognize large cells with striated or twisted nuclei, discrete nucleoli, a broad, acidophilic cytoplasmic border, and low mitotic activity, mixed with a variable number of eosinophils, some cases present mild-to-moderate nuclear atypia [19]. The immunohistochemistry in this case presented positive S100, CD1a, and CD68 markers and negative CD30, discarding other diseases as lymphoma, which was sufficient to confirm the diagnosis (Figure 2). It was not possible to determine the BRAF V600E mutation.

Treatment depends on the classification of the disease. Unifocal cases have a good prognosis and are often curable, requiring local therapy and, depending on the case, surgical resection; if symptoms disappear after a diagnostic biopsy, remission may occur even without treatment [3]. In patients with multisystemic disease and asymptomatic without involvement of critical organs (brain, liver, and lung), an initial period of observation and surveillance is reasonable; treatment should be initiated when symptoms appear. While there is no standard therapy, regimens are based on chemotherapy, and the schemes are based on those observed in pediatric patients, although some schemes have not been validated in the adult population and toxicity has been reported in some case series, requiring treatment to be suspended [3].

Currently used treatments can be classified into two groups: regimens based on vinca alkaloids/steroids and those based on antimetabolites (cladribine and cytarabine) [3]. Although the BRAF-600E mutation is not present in all cases of histiocytosis, the alteration of the BRAF-MEK-ERK axis is universal; BRAF and MEK inhibitors such as dabrafenib and trametinib have recently been investigated for the treatment of patients with high risk or multiorgan damage, with favorable results, although more studies are still necessary to determine its long-term response [20].

In the presented case, it was not possible to initiate treatment, as the patient's empyema could not be eliminated despite undergoing surgery.

## Conclusions

LCH is a rare entity with non-specific symptoms, which makes diagnosis difficult since it is not usually suspected and can simulate other neoplastic diseases, so histopathological study is essential.

Although it can manifest in several organs, it has not been determined that pleural effusion is part of its presentation; however, the pro-inflammatory state generated by the underlying disease may contribute to the appearance of this and other signs that could even mask the diagnosis.

BRAF inhibitors are presented as a possible treatment option for patients for whom chemotherapy is contraindicated, although more research is still needed in this regard.
